# Pyruvate kinase activator: A major breakthrough in the world of Hematology

**DOI:** 10.1016/j.amsu.2022.104631

**Published:** 2022-09-14

**Authors:** Amna Iqbal, Ume Habiba, Radeyah Waseem, Zarmina Islam

**Affiliations:** Dow University of Health Sciences, Department of Medicine, Karachi, Pakistan

**Keywords:** Pyruvate kinase deficiency, Hemolytic anemia, Pyruvate kinase activator, Pregnant and lactating women

## Abstract

Pyruvate Kinase Deficiency (PKD) is a rare genetic disorder targeting Red Blood Cells that manifests as non-spherocytic hemolytic anemia. It has a global distribution with an unknown prevalence, and the frequently reported estimates for different geographical regions show a significant disparity. Because of its hereditary origin, treatment focuses on symptom relief and comfort (principally through blood transfusions, splenectomy, and folic acid supplementation). Pyrukynd (Mitavipat) is a new disease-modifying therapy that was just approved by the FDA and EHA based on clinical trial results that showed a big drop in the number of blood transfusions needed and a rise in hemoglobin levels. The drug stimulates cellular ATP synthesis by acting like Tyrosine Kinase Activator. Even though Pyrukynd has been the subject of studies and is approved for treatment, there is a lack of information on the effects of the medicine on nursing and pregnant mothers. The drug's administration and its effects on minors should also be pleaded.

## 1 Introduction to Pyruvate Kinase Deficiency

Pyruvate kinase deficiency is a rare genetic enzymopathy confined to red blood cells (RBCs) that causes early destruction of RBCs, precipitating as non-spherocytic hemolytic anemia with varying degrees of severity [1]. PKD anemia presents with a conflagration of signs and symptoms in both adults and children, with patients having corresponding hemoglobin levels displaying a unique set of symptoms. In a study by Grace et al. patients reported 38 diverse signs and symptoms, the most prominent ones being yellow eyes and skin, fatigue, emaciation, and shortness of breath [2].

All anemias, including PKD, have a negative impact on the quality of life. Patients with chronic illnesses, such as PK deficiency, occasionally refer to a “new normal” way of life that evolves with acceptance and adaptation to their illness. Grace et al.'s aforementioned study details the 59 detrimental impacts that people with this illness face in a variety of areas, including but not limited to their physical and mental health, daily life activities, and social life [2].

PKD is predominantly passed down in an autosomal recessive manner and has a global distribution with an unknown prevalence, and the frequently reported numbers for various geographical regions demonstrate a substantial discrepancy. The exceptional rarity of the disorder, its phenotypic resemblance to other RBC disorders, its diversified clinical presentation, ethnic and geographic variability, and the lack of adequate data from PK deficiency registries are all factors that contribute to the uncertain prevalence of the disease. In an attempt to find the accurate prevalence and reasons for the variability of the disease, Secrst et al. conducted a systematic review consisting of studies from various parts of the world, such as the United States, the United Kingdom, Canada, Japan, Hong Kong, Africa, Saudi Arabia, Iran, Iraq, Turkey, and India, among others. The review revealed that the disease's general frequency in Western populations is likely higher than the prior estimate of 3.2–8.5 per million, suggesting values as high as 51 per million [3]. Despite the disease's worrying prevalence and profound impact on people's lives, symptomatic treatment had been the norm until the approval of a novel curative drug. This approval has given patients and the scientific community reason for optimism. Nonetheless, it has raised some concerns about the drug's efficacy and safety profile in certain populations, which this brief communication seeks to address.

## 2 Pathophysiology of PKD

Pyruvate Kinase Deficiency is the manifestation of a mutation in the autosomal recessive variants in the PKLR gene located on chromosome 1q21. This leads to a missing or insufficient amount of the enzyme being produced whenever this gene is altered. Pyruvate kinase acts as a key regulator in the glycolytic metabolic pathway by acting as a catalyst and facilitating the conversion of phosphoenolpyruvate to pyruvate, which ultimately leads to the formation of ATP. Glycolysis is an indispensable contributor to ATP production in red blood cells due to their inability to perform aerobic respiration. The abnormal quality or quantity of the PK enzyme leads to altered membrane elasticity and cellular dehydration, making red blood cells prone to premature extravascular hemolysis by the liver and spleen. A lack of enzyme also causes a buildup of upstream products, which ultimately results in an early release of oxygen from hemoglobin [4,5].

## 3 Current guidelines employed in the treatment of PKD

Current guidelines for Pyruvate Kinase Deficiency principally focus on supportive, rather than curative treatment of the disease. After a definitive diagnosis is established by a qualitative and quantitative reduction in enzyme activity and a positive finding of homozygous or heterozygous gene mutations in the PKLR gene, patients are put into supportive care which constitutes the following framework-

1) Folic Acid supplementation - Daily Folic acid supplementation is recommended in patients with moderate hemolysis, or with mild hemolysis coupled with a restricted diet to maintain effective erythropoiesis.
2) Red Cell Transfusions – These should be specified for each patient after a meticulous assessment of their tolerance regarding anemia, quality of life, and physical activity, rather than a measure of their absolute hemoglobin levels. Further assessment after each transfusion is also required.
3) Splenectomy is the definitive treatment in those who are severely anemic or receive regular transfusions and in those at risk of splenic rupture. It is indicated between the age of 5 years to before adolescence. The patient should be properly counseled regarding post-splenectomy sepsis and prophylaxis, and their management [6].

## 4 Pyrukynd (Mitavipat)

A new finding Ongoing research aiming to develop new therapies led to a major breakthrough where Mitapivat was the first approved drug by the FDA and EHA in 2022 as a successful therapy for increasing hemoglobin levels in patients suffering from hemolytic anemia due to PKD. Mitapivat (AG-348) works as a Tyrosine Kinase Activator acting allosterically and thus elevating intracellular levels of Adenosine Triphosphate (ATP) and reducing 2,3-diphosphoglycerate [7]. This has been illustrated in two independent clinical trials, which recruited patients receiving and not receiving blood transfusions respectively. An open-label, clinical trial comprising 27 adult participants, who were on regular blood transfusions due to the stated condition, was conducted and reported a decrease in transfusion burden. The trial was conducted for around 24 weeks. Another Randomized Clinical Trial, lasting about 40 weeks, comprising 80 participants achieved a hemoglobin response in comparison to a placebo. The Pyrukynd tablets up to 50 mg twice daily were administered following an adjustment period [8]. Despite the paramount success of these trials, they are deemed insufficient for the administration of Pyrukynd in pregnant and lactating females.

## 5 Pyrukynd and its limitations in pregnant females

Treatment of anemia in pregnancy is vital to the health of the mother and fetus. It presents as a modifiable, reversible risk factor concomitant with maternal morbidity and mortality. These amalgamate escalating rates of preterm births, pre-eclampsia, low and small for gestational weights, and maternal and perinatal death [9]. Effective management of hemolytic anemia during pregnancy is vital since the rate of hemolysis aggravates during pregnancy. Maintenance of the optimum hemoglobin levels requires regular transfusions which come with an accentuating risk of developing isoimmunization, bloodborne infections, and iron overload. Routine monitoring for hypertension is also highly appreciated [10].

Despite the promising results of Pyrukynd therapy as illustrated, there is meager information available pertaining to the employment of Pyrukynd in pregnant or lactating females secondary to an unevaluated risk of major birth defects, miscarriage, or other fetal or maternal detrimental outcomes. The lack of research in this category has been highlighted by the fact that FDA has not assigned any pregnancy drug category to Mitapivat.

## 6 Future prospects of the drug

Animal studies have been conducted on pregnant rats and rabbits during the period of organogenesis and validated no teratogenic risk up to 13 and 3 times the MRHD (Maximum Recommended Human Dose) respectively. Furthering the experiment, no risk was found in lactating rats at up to 13 MRHD. However, no human trials have been conducted to authenticate these findings in humans [11]. Another limitation of the drug that is yet to be evaluated, is that trials conducted recruited individuals greater than 18 years of age, so the effect on children is still obscure [8].

## 7 Conclusion

The growing popularity and promising results of the clinical trials have, however, raised hope for the treatment of other transfusion-dependent hemolytic anemias, principally Thalassemia which is a much more common ailment afflicting vast numbers globally. A phase 3, randomized, placebo-control trial is underway to explore the possibilities of employing Mitapivat for the treatment of Thalassemia. This will revolutionize the treatment of the disorder and is registered on Cilinicalgtrials.gov under the title of ENERGIZE-T, NCT number NCT04770779 [12].

## Ethical approval

This paper did not involve patients, therefore no ethical approval was required.

## Please state any sources of funding for your research

No funding was acquired for this paper.

## Author contribution

Amna Iqbal: Conceptualization, Writing – original draft, final approval and agreeing to the accuracy of the work, Ume Habiba: Writing – original draft, final approval and agreeing to the accuracy of the work. Radeyah Waseem: Writing – original draft, final approval and agreeing to the accuracy of the work, Zarmina Islam: Writing - review & editing, final approval and agreeing to the accuracy of the work.

## Please state any conflicts of interest

The authors declare that there is no conflict of interest.

## Registration of research studies

1. Name of the registry: Not Applicable.
2. Unique Identifying number or registration ID: Not Applicable.
3. Hyperlink to your specific registration (must be publicly accessible and will be checked): Not Applicable.

## Guarantor

Amna Iqbal, Ume Habiba, Radeyah Waseem, Zarmina Islam

## Consent

This study was not done on patients or volunteers, therefore no written consent was required.
